# Establishing the A. E. Watkins landrace cultivar collection as a resource for systematic gene discovery in bread wheat

**DOI:** 10.1007/s00122-014-2344-5

**Published:** 2014-07-02

**Authors:** Luzie U. Wingen, Simon Orford, Richard Goram, Michelle Leverington-Waite, Lorelei Bilham, Theofania S. Patsiou, Mike Ambrose, Jo Dicks, Simon Griffiths

**Affiliations:** John Innes Centre, Norwich Research Park, Norwich, UK

## Abstract

**Key message:**

**A high level of genetic diversity was found in the A. E. Watkins bread wheat landrace collection. Genotypic information was used to determine the population structure and to develop germplasm resources.**

**Abstract:**

In the 1930s A. E. Watkins acquired landrace cultivars of bread wheat (*Triticum aestivum* L.) from official channels of the board of Trade in London, many of which originated from local markets in 32 countries. The geographic distribution of the 826 landrace cultivars of the current collection, here called the Watkins collection, covers many Asian and European countries and some from Africa. The cultivars were genotyped with 41 microsatellite markers in order to investigate the genetic diversity and population structure of the collection. A high level of genetic diversity was found, higher than in a collection of modern European winter bread wheat varieties from 1945 to 2000. Furthermore, although weak, the population structure of the Watkins collection reveals nine ancestral geographical groupings. An exchange of genetic material between ancestral groups before commercial wheat-breeding started would be a possible explanation for this. The increased knowledge regarding the diversity of the Watkins collection was used to develop resources for wheat research and breeding, one of them a core set, which captures the majority of the genetic diversity detected. The understanding of genetic diversity and population structure together with the availability of breeding resources should help to accelerate the detection of new alleles in the Watkins collection.

**Electronic supplementary material:**

The online version of this article (doi:10.1007/s00122-014-2344-5) contains supplementary material, which is available to authorized users.

## Introduction

Hexaploid bread or common wheat (*Triticum aestivum* L.) is an important staple crop with over 600 million tonnes being harvested annually. Wheat was originally domesticated about 10,000 years ago in the fertile crescent (see Shewry 2009 for a review). The wheat genome has derived from hybridisation of the domesticated tetraploid progenitor emmer (*Triticum dicoccoides*), the donor of the A and B genome, with the wild diploid species *Aegilops tauschii*, the donor of the D genome (Salamini et al. 2002). From its origin of domestication, which is located in today’s southeastern part of Turkey, the crop was spread by the human population and cultivated in many parts of the world. It came to Europe via a route to Anatolia, then to Greece. From there, one way proceeded northward through the Balkans to the Danube. A second route went across to Italy, France and Spain, finally reaching UK and Scandinavia. In a similar way, wheat spread via Iran to central Asia, reaching China, and via Egypt into Africa. It was introduced by Spaniards to Mexico in 1529 and to Australia in 1788 (Feldmann 2001).

Domestication has reportedly introduced population bottlenecks leading to a lower genetic diversity in crop plants in comparison to wild ancestors (Doebley et al. 2006). Following the introduction of domesticated wheat, varieties became adapted to local conditions becoming the so-called landrace cultivars (LCs). In this process, the genetic variation was further reduced by genetic drift and selection (Reif et al. 2005). However, the process is unlikely to have happened under complete isolation, rather exchange of breeding material between neighbours and by more distant trade will have occurred. Subsequently, genetic diversity of modern elite cultivars (MC) may have even become lower, if breeding was based on a narrowing germplasm base (Tanksley 1997). A recurrent theme in this research is the possibility that more diversity may have been left behind in LCs during the ‘green revolution’ when breeding strategies hypothetically focused on a few target genes only (Khush 2001). However, this assertion is not generally true. A narrowing or enrichment of the germplasm diversity depends on the particular breeding programme applied. Among CIMMYT wheat varieties in the period from 1949 to 1989 a decrease in genetic diversity was found, contrary to an increase in the period from 1990 to 1997 (Reif et al. 2005). Different trends for the genetic diversity of modern wheat lines in the USA (increasing), Australia (constant) and the UK (decreasing) between 1940 and 2005 have been reported (White et al. 2008). Similarly, genetic diversity trends could be linked to wheat breeding practices in European countries (Roussel et al. 2005), where MCs from western countries show a lower number of alleles. Furthermore, it has been shown that the narrowing of the germplasm base in bread wheat can be averted through the introgression of novel materials, e.g. coming from LCs (Smale et al. 2002; Reif et al. 2005).

A. E. Watkins, from the School of Agriculture in Cambridge, was interested in Vavilov’s work on the origins of crop plants and plant domestication (Watkins 1933; Vavilov 1931). He thus acquired bread wheat and macaroni wheat (*Triticum durum* Desf.) LCs from markets predominantly in Asia and Europe, but also from other parts of the world using connections with the London Board of Trade. In this way, he established a considerable wheat collection of over 7,000 accessions (Miller et al. 2001). Unfortunately many were lost during the Second World War when the material was put into storage. The collection today consists of a current viable bread wheat LC collection of 826 accessions, here called the Watkins collection. The Watkins collection captures a snap shot of genetic diversity present before the start of modern breeding. This is compared to the diversity present in MCs adapted to Northern European climate. The EU Commission Key Action 5 project ‘Genetic Diversity in Agriculture: Temporal Flux, Sustainable Productivity and Food Security’ (Gediflux) was established to investigate the impact of intensive breeding over time on the genetic diversity of different European crops, one of them winter bread wheat (Reeves et al. 2004). A panel of over 500 wheat MCs from across Europe, here called the Gediflux collection, which had been sown in major acreages in the years 1945–2000, has been used for this study. The panel was genotyped using 42 microsatellite (single sequence repeat, SSR) markers and the genetic diversity was assessed (Reeves et al. 2004). In general, no significant change in genetic diversity was detected over time for any of the crops studied, including the winter bread wheat panel.

The threat of climate change and the growing human population paired with the observed lower rates of genetic gain calls for improved methods to obtain a sustainable increase of crop yields. Increasing crop diversity by exploiting the diversity of LCs is one strategy to approach this goal. Useful and currently rare alleles, introgressed into elite wheat lines, may help to improve grain yield or to adapt the plants to new climate conditions. Studies on the genetic diversity of bread wheat LC collections (Huang et al. 2002; Balfourier et al. 2007; Horvath et al. 2009) reveal a high level of genetic diversity and suggest a rich source of alleles not used in modern breeding.

The Watkins collection has been successfully used to find new alleles or genes for leaf rust resistance (Dyck 1994), stripe rust resistance (Bansal et al. 2011), and root-lesion nematode resistance (Thompson and Seymour 2011). However, all of these are Mendelian traits, and the determination of the chromosomal locations for such traits is comparatively easy. A better genetic understanding of LC collections is necessary in order to dissect the architecture of complex traits.

In order to improve allele mining in the Watkins collection it was genotyped with a set of 41 SSR markers, which was partly overlapping with the set used for the Gediflux collection. The current study reports on the results of phenotyping and genotyping, the diversity of the Watkins collection and its genetic population structure. Comparisons to other bread wheat collections, particularly the Gediflux collection, are presented. Furthermore, the development of breeding resources, particularly of a core set of LCs, is reported.

## Materials and methods

### Plant material, growing conditions, and phenotyping

Seeds for the Watkins collection were received from the John Innes Centre Germplasm Resource Unit (GRU http://www.jic.ac.uk/germplasm/). Single seed descendents (SSDs) were developed over 6 generations for all 826 LCs. Initially, four seeds were sown for each LC. In 234 cases, phenotypes from the same accession showed striking differences in the first generation and, thus, two SSD streams were produced from those accessions, here called sister lines. All field trials were grown at Church Farm Bawburgh, Norwich, UK (52.63°N, 1.18°E), under standard growing conditions, if not stated differently. Trials with replicates were planted in a randomised block design The majority of SSDs, developed from original accessions of the Watkins collection, were planted in 2006 in four replicates of 1 m × 1 m field plots without nitrogen fertiliser usage. The following traits were measured: mature plant height, ear emergence, grain yield, lodging, vernalisation requirement, presence of awns, thousand grain weight, grain length, grain width and grain surface area. The Watkins core set (119 LCs) and the Gediflux collection were grown in 2011 in field plots of 1.5 m × 4 m (single plots) and 1 m × 1m (three replicates), respectively, and assessed for mature plant height (Gediflux only), peduncle and internode lengths (Gediflux only), ear emergence, grain yield, thousand grain weight, grain length, grain width and grain surface area. A trait mean was calculated for the varieties of the core set for each trait and both years, 2006 and 2011, with exception of the Watkins 2011 data, where only single plots were measured. For comparison of phenotypic observations between those years, the 2006 data set values were adjusted to the 2011 level. Bi-parental SSD populations were developed for several LC accessions. For this the elite wheat cultivar ‘Paragon’ was crossed with selected Watkins LCs followed by four rounds of self pollination. The selection criterion for the LC parent was the display of a phenotype within the extreme borders of the phenotype range. Bi-parental populations were grown in 2011 for multiplication purposes in single 1 m × 1 m field plots and assessed for plant height and flowering times.

### Genotyping

Genomic DNA was extracted from 3-week-old seedlings using the DNeasy 96 Plant Kit and protocol for fresh plant tissue (Qiagen). The genotyping of the Watkins collection was conducted using 41 publicly available SSR markers. Public primer sets were used from JIC (psp), IPK Gatersleben (gwm/gdm), Wheat Microsatellite Consortium (wmc), Beltsville Agricultural Research Station (barc) and INRA (cfd/cfa) collections, and can be found on the GrainGenes website (http://www.wheat.pw.usda.gov/). Targeted markers were selected to fall on different chromosomes according to a published consensus map (Somers et al. 2004). Initial marker tests on a limited number of varieties helped to identify markers that exhibit scorable, multiple alleles. These markers were given preference. See Table 1 for the names of the markers. There were 14 common markers between the SSR marker set used here and the markers employed on the Gediflux collection.

Gene-based assays were conducted: the presence of the recessive vernalisation requirement alleles were tested with allele specific assays for *Vrn-A1* [three assays (Yan et al. 2004)], *Vrn-B1* and *Vrn-D1* [two assays each (Fu et al. 2005)]; the presence of the recessive photoperiod sensitivity alleles were tested with allele specific assays for *Ppd-A1c.1* [one assay (Wilhelm et al. 2008)], *Ppd-B1a.1-3* [three assays (Díaz et al. 2012)] and *Ppd-D1.a1* and *Ppd-D1.c2* [two assays (Beales et al. 2007)].

Forward primers were labelled with the dyes FAM, VIC, NED, or PET (Applied Biosystems) according to Schuelke (2000). PCR mixes were in 6.25 µl volumes that consisted of 3.125 µl HotstarTaq Master Mix (Qiagen), 0.75 µM of each primer, and 12.5 ng gDNA. The PCR profile consisted of 15 min at 94 °C, followed by 35 cycles of [95 °C for 1 min, a primer pair-dependent annealing temperature according to the GrainGenes website for 1 min, and 72 °C for 1 min], and concluded with 72 °C for 10 min. Products were measured on an ABI 3730 DNA Analyzer with a POP-7(TM) polymer column. Peak data were analysed using the manufacturers GeneMapper (version 4.0) software.

Reactions that did not show an amplified product were repeated. A NULL allele was scored, if: (a) the repeat did not result in a PCR product; (b) the DNA quality was good, as could be seen from the scores of other markers; and (c) the number of missing amplifications for that marker was lower than 5 %. Otherwise, the data point was scored as missing.

Of the 41 SSR markers, 39 markers had a good score with less than 5% missing values after one round of re-genotyping of missing calls. Some of the markers detected more than one locus. If a clear separation between loci could be made, the marker was scored multiple times, otherwise only scores for the most consistent locus were taken. This resulted in genotypic information for 45 loci (see Table 1). The number of missing data per locus was on average 4.2, including eight loci with no missing data but with NULL alleles, and three loci with more than 20 % missing data. The latter loci were excluded from the advanced statistical analyses. Of the final marker set 14 markers were shared with the Gediflux collection.

**Table 1:** Summary of genotyping outcome and diversity statistics of the Watkins collection for 41 SSR markers binding to 45 loci in the bread wheat genome and six gene-based markers. Equations for diversity indices are given in Table S1. Mean and range are given over SSR marker loci only

Marker name	chr	Missing	*d*_AR_	*d*_RAR_	$d_{\hat{r}(g)}$	*d*_Nei_	*d*_PIC_	*d*_SWI_
barc019	3A	0.0	16	8	7.9	0.74	0.71	1.71
barc021	7A	2.7	16	6	8.4	0.58	0.57	1.46
barc029	7A	1.7	12	6	5.8	0.61	0.57	1.28
barc032	5B	0.9	16	5	9.9	0.83	0.81	2.06
barc096	6D	0.2	16	12	5.1	0.53	0.47	1.07
barc097	7BD5B	16.9	11	6	5.8	0.74	0.70	1.52
barc107	6A	0.9	14	8	6.2	0.50	0.46	1.12
barc110	5D	0.3	29	14	14.9	0.93	0.92	2.75
barc127	6B	0.4	19	12	8.2	0.83	0.81	1.97
barc134	6B	11.2	20	12	9.0	0.80	0.78	1.93
barc164	3B	0.1	29	16	13.3	0.86	0.85	2.42
barc172	7BD5B	0.0	11	3	6.8	0.77	0.74	1.67
barc240	1ABD5B	0.1	33	18	13.1	0.85	0.83	2.37
cfd079.a	3ABD	8.4	26	9	14.5	0.91	0.90	2.65
cfd079.b	3ABD	14.0	13	8	6.5	0.74	0.70	1.59
gdm111	1D	7.8	13	5	7.3	0.75	0.71	1.64
gdm129	4D	0.0	8	5	3.4	0.22	0.21	0.49
gwm003*	3D	0.0	16	9	7.2	0.69	0.66	1.56
gwm018*	1B	0.6	15	7	7.8	0.74	0.71	1.69
gwm030.a	2D3A	23.2	16	8	9.0	0.79	0.77	1.91
gwm030.b	2D3A	10.4	28	9	16.9	0.93	0.93	2.88
gwm046*	7B	1.6	28	10	15.1	0.91	0.91	2.71
gwm095*	2A	0.0	14	7	7.7	0.81	0.79	1.86
gwm155*	1D3A	0.5	24	13	10.1	0.80	0.78	2.01
gwm190*	5D	0.6	22	12	9.9	0.84	0.83	2.12
gwm213*	5B	1.5	46	19	23	0.96	0.95	3.38
gwm219*	6B	0.0	30	11	16.0	0.91	0.91	2.76
gwm291*	5A	13.0	29	12	15.0	0.87	0.86	2.56
gwm312*	2A	0.1	43	25	17.3	0.89	0.88	2.80
gwm337	1D	3.3	25	11	12.6	0.88	0.87	2.43
gwm357*	1A	0.1	11	4	6.1	0.73	0.69	1.53
gwm437*	7D	0.0	26	8	16.3	0.93	0.93	2.83
gwm456*	3D	0.1	18	7	10.1	0.84	0.82	2.10
gwm526.a	2B	3.7	7	3	4.2	0.62	0.58	1.18
gwm526.b	2B	3.5	15	6	8.5	0.77	0.74	1.80
gwm539	2D	0.4	61	34	23.5	0.96	0.96	3.45
gwm570*	6A	0.0	26	14	12.7	0.89	0.88	2.45
gwm608.a	2D4D6B	21.4	6	0	6.0	0.80	0.77	1.70
gwm608.c	2D4D6B	0.7	20	9	11.3	0.89	0.88	2.38
psp3100	1B	0.6	54	36	17.6	0.92	0.92	2.95
wmc093	1AD	1.1	9	7	2.7	0.51	0.39	0.78
wmc105	6B	4.1	40	20	18.5	0.91	0.90	2.90
wmc110	5A	22.4	3	0	2.7	0.34	0.29	0.59
wmc154	2B	0.6	38	28	11.5	0.78	0.76	2.11
wmc168	7A	12.3	34	14	15.3	0.80	0.79	2.40
mean (SSRs)		4.2	22.4	11.0	11.1	0.78	0.75	2.09
min (SSRs)		0.0	3	0	2.7	0.22	0.21	0.49
max (SSRs)		23.2	61	36	24.9	0.96	0.96	3.45

*Ppd-A1*	2A	3.4	3	1	1.2	0.14	0.13	0.29
*Ppd-B1*	2B	1.8	5	2	1.4	0.23	0.22	0.49
*Ppd-D1*	2D	0.0	7	2	2.0	0.61	0.56	1.16
*Vrn-A1*	5A	9.7	5	2	1.2	0.16	−1.25	0.4
*Vrn-B1*	5B	18.6	4	0	1.9	0.56	0.45	1.00
*Vrn-D1*	5D	18.1	4	1	1.5	0.29	0.27	0.60

Markers shared with Gediflux collection are indicated by a^*^ after the marker name

*chr* putative chromosomal locations according to Gramene database (http://www.gramene.org/markers), *d*_*AR*_ allele richness,* d*_*RAR*_ number of rare alleles, $d_{\hat{r}(g)}$ allele richness after rarefaction, *d*_*Nei*_ Nei’s gene diversity, *d*_*PIC*_ polymorphic information content, *d*_*SWI*_ Shannon–Weaver Diversity Index

### Statistical analysis

#### Diversity statistics

The genetic diversity of a collection of cultivars was calculated using R software (vs. 3.02) (R Core Team 2013) for different common diversity indices (compare Table S1 for equations). The Shannon–Weaver Diversity Index on phenotype scores (*d*_SWIp_) was calculated when traits were measured in all three trials. For this, the overall phenotype range was divided into 12 phenotype classes of similar size. For each trial, the frequencies of scores in each class were determined. d_SWIp_was calculated similar to d_SWI_(see Table S1) from these frequencies.

#### Population structure

To investigate the population structure of the Watkins and the Gediflux collections, the Bayesian model-based clustering method implemented in the programme STRUCTURE (Pritchard et al. 2000) was used. The full dataset from 1,054 lines was used, including all sister lines where present (234 cases). Settings for STRUCTURE were: admixture, burn-in period of 10,000 and runs of 50,000 steps. Runs for numbers of founder populations between two and 25 were performed with 10 repetitions each. The number of ancestral clusters was determined by the *δK* statistic (Evanno et al. 2005) using R software and package corrsieve (vs. 1.6–8).

### Core set

A reduced set of LCs was initially selected from the diverse phenotypes. The diversity of the core was determined using the CoreHunter software (Thachuk et al. 2009) for different diversity indices: Cavalli-Sforza and Edwards Distance, Modified Rogers Distance, Number of Effective Alleles Index, and Shannon–Weaver Diversity Index (*d*_SWI_). For the final selection of the core set of accessions the *d*_SWI_ was used, as the highest value of the genetic diversity averaged over the four diversity indices was achieved for this index.

## Results

### Phenotypic diversity

The phenotypic diversity of the Watkins collection has been assessed in field trials for the majority of LCs (726 accessions). Scores for the traits adult plant height, heading date, and four grain characteristics were taken (Table S2). A wide range of phenotype scores were found for the traits scored, as indicated by high diversity values (Shannon–Weaver phenotype diversity scores (*d*_SWIp_) between 1.37 and 2.03). Watkins LCs with extreme phenotypes for any of the above traits were selected as parents for the development of bi-parental populations (Table S2). The collection was also scored for vernalisation requirement: 86% of the lines showed spring growth habit, and only 14% winter growth habit.

**Fig. 1:**
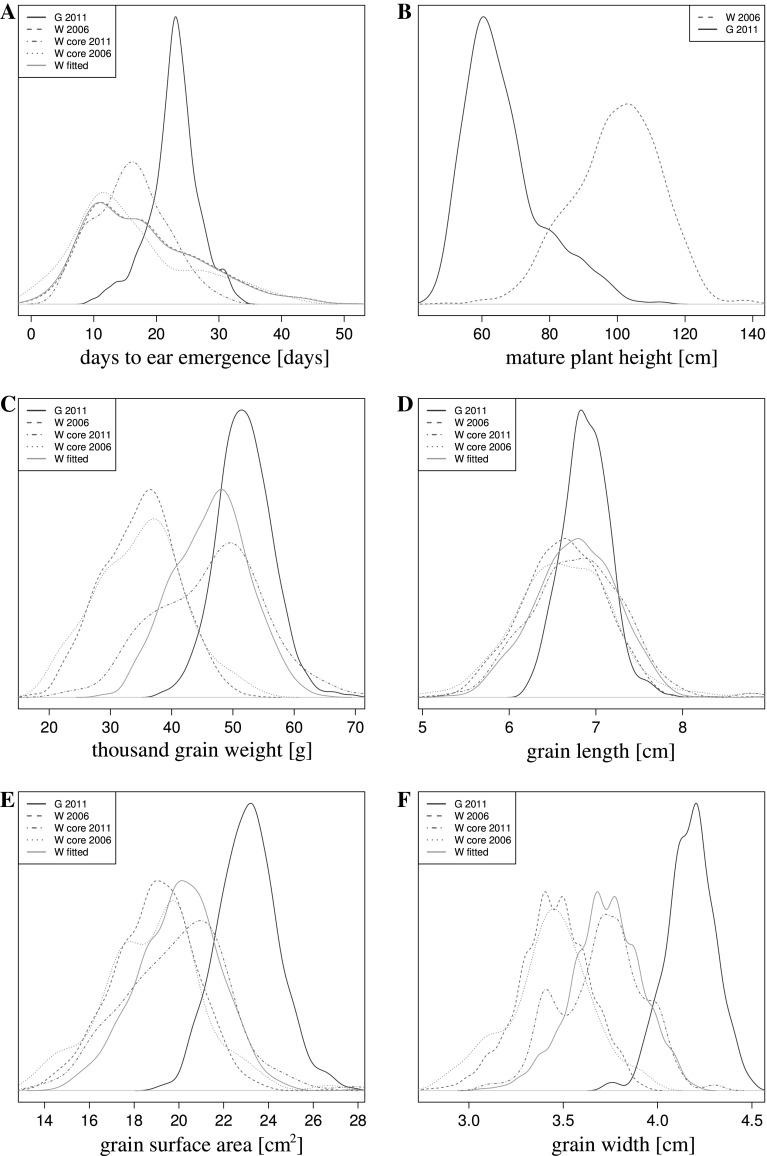
Outlines of density functions created from phenotypic values for the following bread wheat collections or sets: Watkins 2006 (*red*, *hashed*); Gediflux 2011 (*blue*); Watkins core 2006 and 2011 (*red*, *dotted* and *dot hashed*, respectively); Watkins data fitted to 2011 conditions (*orange*). **a** Days to ear emergence [days after May 1st], **b** plant mature height [cm], **c** thousand grain weight [g], **d** grain length [cm], **e** grain surface area [cm^2^], **f**: grain width [cm]. Abbreviations: *W* Watkins, *G* Gediflux, *2006 and 2011* years collections were grown (colour figure online)

In general the phenotypic variation observed in the Watkins collection was larger than that observed in a collection of European MCs, the Gediflux collection as indicated by *d*_SWIp_ values between 0.97 and 1.74 for the same traits as measured for the Watkins collection (see Fig. 1 and compare Tables S2 and S3). The two collections displayed different trends for most of the traits. In the Gediflux collection the window of flowering times was smaller than in the Watkins collection. Similarly, in the Gediflux collection the mature plant height was lower, whereas the average thousand grain weight was high. As an exception in the traits observed, grain length values did not show a very different distribution between the two collections. In contrast, grain surface area and grain width were both higher in the Gediflux collection.

### Genetic diversity

The genotypic scores on 45 loci reveal an average allele number per locus is 22.4, ranging from 3 to 61 alleles. The allele numbers for individual markers are listed in Table 1. The average d_Nei_ is 0.78, ranging from 0.22 to 0.96, the average *d*_PIC_ is 0.75, ranging from 0.21 to 0.96, and the average *d*_SWI_ is 2.09, ranging from 0.49 to 3.45. The diversity ranking of the markers is in general similar for the diversity indices tested, with markers gwm539 and gwm213 being the most diverse. This indicates that all the diversity indices used were able to identify the genetic diversity present.

A comparison between SSD lines stemming from the same original LC accession but which showed phenotypical heterogeneity (234 cases or 28% of accessions) was conducted. This showed that on average 15.7 (35.7 %) of the markers had different allele sets between sister lines (SD 10.6 markers, range 1–39 markers). This gives some indication of the heterogeneity of the original LC accessions.

Using the genotype data generated by the Gediflux project (Reeves et al. 2004), we were able to compare the diversity of the LC collection to a modern wheat collection. In the Gediflux collection, the average *d*_PIC_ value is just 0.57 (see Table S4), much lower than that found in the Watkins collection (0.75). Similar observations can be made for Nei’s gene diversity (*d*_Nei_=0.62 vs. 0.78) and the Shannon–Weaver Diversity Index (*d*_SWI_=1.30 vs. 2.09). Detailed results can be found in Table S4.

In order to understand the origin of the diversity better, the diversity of the three different wheat genomes were determined separately, by only using markers specific for single genomes. Average diversity values for A, B and D genome markers for the Watkins collection were found as *d*_SWI_: 1.79, 2.30 and 1.93 and *d*_PIC_ : 0.68, 0.81 and 0.71, respectively. Values for the Gediflux collection are *d*_SWI_: 1.14, 1.47 and 1.07 and *d*_PIC_: 0.54, 0.68, 0.56, for the three different genomes, respectively. In both cases this suggests that the B genome is more diverse than the two other genomes. However, this analysis is based on 11, 13 and 11 markers, respectively, for the A, B and D genome in the Watkins collection only, and on 11, 14, and 10 markers, respectively, in the Gediflux collection. A simple bias due to analysing different marker numbers from each genome was excluded by calculating the mean diversity index from eight markers randomly selected from each genome, which resulted in near identical values (Watkins: *d*_SWI_: 1.72, 2.25 and 1.95 and *d*_PIC_: 0.67, 0.81 and 0.72 Gediflux: *d*_SWI_: 1.16, 1.43 and 1.07 and *d*_PIC_: 0.51, 0.62 and 0.50, respectively).

### Population structure of the Watkins collection

**Fig. 2:**
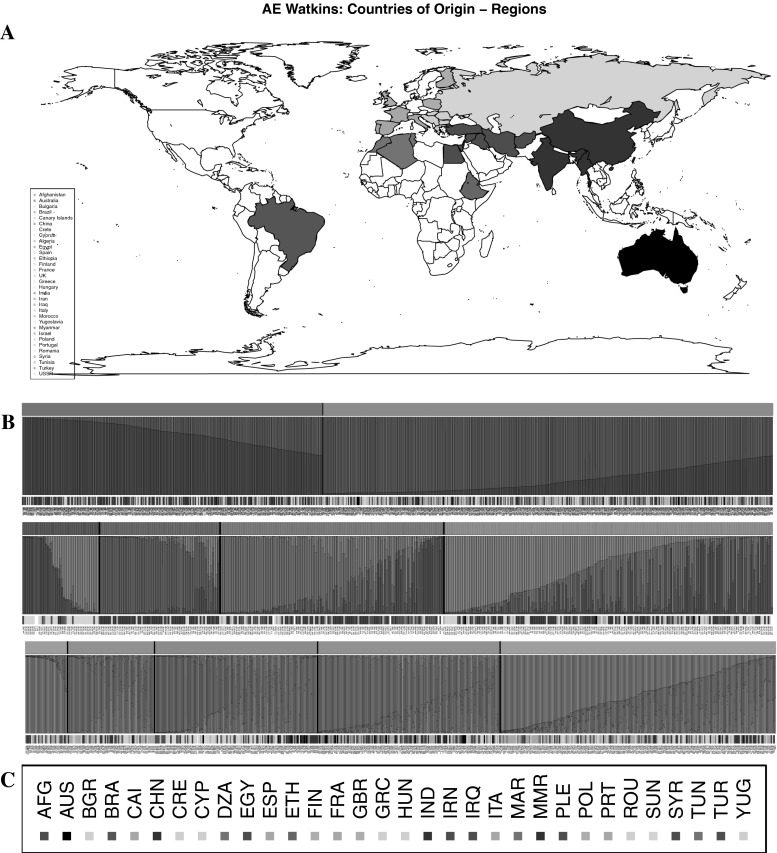
**a** The world map. Countries from which LCs were acquired are *coloured*. *Colours* are organised in geographic regions. **b** STRUCTURE assignment of the Watkins LCs to ancestral populations. Three panels shown. *Top panel* whole collection; *middle and lower panel* the 424 and 630 subpopulations of the whole collection, respectively. Each panel is divided into three rows. *Top row* assignment to ancestral population; *middle row* ancestral characteristics of each line; *bottom row* colour code of country/region of origin. Abbreviated names of the LCs are given below the* bottom row*. **c** Colour code of the countries, according to geographic regions

An analysis to determine the population structure of the Watkins collection by Bayesian model-based clustering (Pritchard et al. 2000) was undertaken, using the SSR genotype data. The number of ancestral groups was determined by *δK* statistics (Evanno et al. 2005). This analysis indicates a split of the collection into two ancestral groups or subpopulation (Fig. S1B). 85% of sister lines fell into the same subpopulation. A total of 424 of the accessions show more characteristics of group 1 and 630 of group 2. Although more Asian LCs are found in group 1 and more European LCs are in group 2, on the whole the groups are composed of accessions from different geographic regions. A further analysis was conducted on the next hierarchical level. This analysis addressed the structure within each of the two subpopulations and revealed that the smaller group was most likely formed from four ancestral subpopulations. The structure of the larger group is more obscure, but five being the most likely number of ancestral subpopulations (Fig. S1C and D, respectively). These groupings were aligned with the geographic region from which the LCs were collected, as shown in Fig. 2. Geographic origins could then be assigned from this alignment as follows: a Russian (group 1.1), a Chinese/Indian (group 1.2), a Central/ East Asian (group 1.3), and a mixed European/Asian (group 1.4) group form the 424 LCs subpopulation. The 630 LCs strong subpopulation appear to comprise a South European/Asian (group 2.1), a Northwest European (group 2.2), an East European (group 2.3), a South Mediterranean/African (group 2.4), and a North Mediterranean (group 2.5) group.

### Population structure of the Gediflux collection

**Fig. 3:**
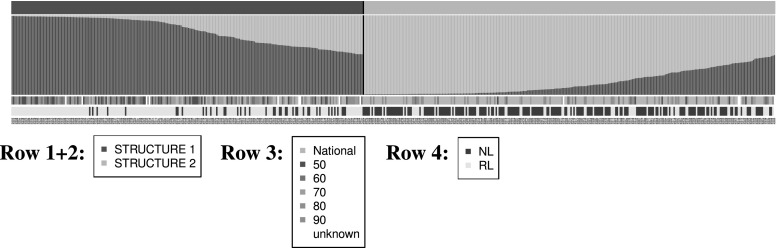
Representation of the STRUCTURE assignment of the Gediflux MCs into two ancestral populations. The panel is divided into four rows which show (from* top* to* bottom*): *Row 1* assignment to ancestral population as detected by STRUCTURE (population 1: *dark green*, population 2: *light green*). *Row 2* ancestral characteristics of each line as detected by STRUCTURE (*colours* as in* row 1*). *Row 3* colour code of decade of release for varieties from the EU recommended list. *Row 4* colour code of origin: EU recommended list (*green*) or UK national list (*yellow*). The accession numbers of the Gediflux varieties are given below* row 4*. Legends with colour code for the different rows are given below the plot (colour figure online)

The determination of the population structure of the Gediflux collection by Bayesian model-based clustering, similar to the analysis performed for the Watkins collection, revealed a main subdivision of the population into two clusters (Fig. 3, top panel, and Fig. S2). These ancestral subpopulations were mainly supported by accessions either coming from the EU recommended list or from the UK national list, respectively. The analysis did also hold some support for the presence of 15 ancestral populations. (Fig. S2 B). The differences between these groups seem to be a combination of the decade of breeding and the geographic region, UK or EU. A further hierarchical analysis was not undertaken.

### Core set

A core set of LCs from the Watkins collection was chosen to preserve the majority of the genetic diversity while reducing the numbers of LCs necessary to conduct trials. The selected core set contains 119 LCs and preserves 98 or 96% of the total genetic diversity, as detected by diversity measurements for the employed markers, *d*_Nei_ or *d*_SWI_, respectively. However, the number of alleles, particularly of rare alleles, is strongly reduced in the core set as can be seen from the *D*_AR_ and *D*_RAR_ values in Table 1 and S6. This could mean that the Core Set is not a suitable tool to identify very rare alleles. A detailed list of the accessions included in the core set can be found in Table S5 and the genetic diversity levels are summarised in Table S6.

## Discussion

The present study reports on the phenotypic and genotypic diversity of the Watkins bread wheat LCs collection of 826 accessions. Regarding the former, a scoring of phenotypic values for basic traits was conducted. The phenotypic variation observed in the Watkins collection was larger than that observed in a collection of European MCs, the Gediflux collection (compare Table S2 and S3 and Fig. 1). For most of the traits a clear trend between the two collections was observed. These trends will be the result of modern breeding strategies, which were employed in the development of MCs. Partly, trends will also reflect the differences in geographic distribution. The Gediflux collection of winter wheat was adapted for a narrow geographic region, Northwestern Europe, in comparison to the Watkins collection, which encompasses a near-global scale. Traits with a low genetic variability are not expected to show a trend. Trends were observed for plant height, flowering time and several grain characteristics, but not for grain length. A low genetic variability for this trait must be assumed. The window of flowering times was narrower in the Gediflux collection, and the mature plant height was reduced. These characteristics make the plants more adapted to modern farming under European growing conditions. In contrast, the average thousand grain weight, grain surface area and grain width were all increased in the modern collection, which seems likely to be a result of modern breeding for higher yields via larger seeds (Gegas et al. 2010).

A spring growth habit under the North European spring sowing conditions was observed for 86% of the Watkins LCs studied. Of those LCs which showed spring growth habit, 178 accessions (24%) did not carry a spring type alleles at any of the three *Vrn-1* loci. This may be partly due to the UK conditions allowing weak winter types to get vernalised due to cold nights in March. However, given the high number of cases, this suggests that another pathway leading to a spring growth habit may be responsible in some of those LCs. Up to five vernalisation genes have been reported for winter wheat to date, but so far mainly the three *Vrn-1* homeologues have been well investigated (Distelfeld et al. 2009). The identification of LCs with *Vrn-1*-independent spring growth habit could help to discover more details of the vernalisation pathway in wheat. The genetic diversity found for the Vrn-1 and Ppd-1 genes was low. This is expected as these genes were most likely under selection as they play a major role in adaptation to the local climate. Due to selection pressure, the number of alleles would be low in the progenitor plants. Moreover, the genotyping using gene-based markers only revealed the presence of known alleles. Further alleles present, will not be detected.

The genotyping of the Watkins collection, using 41 SSR markers was followed by an analysis of the genetic diversity levels present at the marker loci. A high level of genetic diversity was detected irrespectively of the index of diversity used (see Table 1). An average allele number of 22.4 and a gene diversity index (*d*_PIC_) of 0.75 were found. These values are at the higher end of those found in other wheat LC collections (see Table S7 for a list of published studies). The genetic diversity of the IPK bread wheat collection of 998 LCs from 68 countries was reported as an average allele number of 18.1 per locus and a *d*_PIC_ of 0.77 (Huang et al. 2002) (see also Table S7). The INRA collection (nearly 4,000 cultivars) revealed an average allele number of 23.9 per locus scored and a *d*_PIC_ of 0.74 (Balfourier et al. 2007). The genetic diversity values found for the three LCs collections are of similar levels, although they were not found with the same marker set. They can therefore be seen as a rough guide when comparing the diversity levels of the populations. A further insight can be gained by looking at shared markers only. Eight markers were shared between the study presented here and the IPK study (Huang et al. 2002). In general, the same level of allele number and gene diversity are found for those eight markers (see Table S8) in both studies. The analysis presented also possessed eight markers in common with the INRA study Balfourier et al. (2007). In this case more discrepancies between the diversity levels detected are found (see Table S8). However, a less strict correlation was expected, as MCs were included in the INRA collection. Also, the number of INRA accessions analysed was five times higher than in the Watkins collection. INRA LCs came from a greater spread of countries. The differences between the studies could reflect the genetic diversity present in those additional geographic regions, which are not present in the Watkins collection.

The diversity levels found in the Watkins collection were also compared to those of the Gediflux collection, which stands for the type of Northern European germplasm breeding would be targeted for. It seems apparent from this comparison that the LCs assembled in the Watkins collection preserve a much higher level of genetic diversity than present in the MCs. The gene diversity (d_Nei_) values are 0.78 versus 0.65 for the Watkins and the Gediflux collections, respectively. A more detailed comparison of the diversity levels for single markers was possible, due to 14 markers being shared between the studies. The Watkins diversity values were consistently higher for all 14 markers (see Table S9).

In the present study we found the diversity of the B genome to be higher than those of the A and D genomes. The differences in diversity between the three ancient wheat genomes deviates from that found in wheat SNP discovery approaches, which find the D genome being less diverse than the A and B genomes (Allen et al. 2011; Cavanagh et al. 2013). In particular, no difference in diversity between A and D genome was detected, whereas the A genome should show a higher diversity than the D genome. This lack of discrimination is potentially due to a bias in marker choice, where diverse markers were given a preference. Furthermore, the presented analysis was based on a small number of markers only, and may thus not robustly represent the diversity of the different genomes.

### Population structure

The grouping of the Watkins collection into two major ancestral populations and the subsequent hierarchical subdivision into four and five ancestral subpopulations for the two groups, respectively, was detected by Bayesian model-based clustering (see Fig. 2). Accessions of the ancestral subpopulations could be predominantly assigned to the following geographic regions: Russia (group 1.1); China/India (group 1.2); Central/East Asia (group 1.3); mixed Europe/Asia (group 1.4); South Europe/Asia (group 2.1); Northwest Europe (group 2.2); East Europe (group 2.3); South Mediterranean/Africa (group 2.4); and North Mediterranean (group 2.5) (see Fig. 2).

The geographic regions found here are in principle in good agreement with other studies of bread wheat LC collections. Five ancestral groups were found within a 372 accession strong core set of the INRA bread wheat collection using markers for chromosome 3B (Horvath et al. 2009). A division into Northwest Europe, Southeast Europe, Asia, Nepal and CIMMYT-ICARDA is reported. The Watkins collection does not contain samples from Nepal and CIMMYT-ICARDA, so those ancestral groups cannot be discovered in the present analysis. However, a split between Northwest Europe, Southwest Europe and Asia was detected in the Watkins population. An analysis of just 235 of the above 372 accessions with 82 SSRs, covering the whole genome (Rousset et al. 2011) again reports five, but slightly different groups. The groups are: Northwest Europe, Southeast Europe, Mediterranean, Central Asia and South America/Africa. Here the group from Nepal is not identified but a separate Mediterranean group was found. Several of the groups found are also present in the Watkins collection. A Northwest European (group 2.2), East European (group 2.3), and a Mediterranean-dominated group (groups 2.4 and 2.5) are found for both studies. However, a further small but distinct European–Asian Group (group 2.1) was found in the Watkins collection alone. Moreover, several Asian groups (groups 1.2–1.4) and the Russian group (group 1.1) were only found in the Watkins collection. The INRA collection contains a small number of Asian accessions (Balfourier et al. 2007; Rousset et al. 2011) in comparison to the Watkins collection (over 300 LCs). The detected Asian cluster is thus the smallest cluster in the INRA analysis. Not surprisingly no subdivision of that cluster was detected, most likely due to scarcity of genetic information. The Watkins collection, however, contains a large number of Asian LCs, and several ancestral groups from Asia are revealed. This shows that the Watkins collection has good additional value to the other LC collections. Regarding the large number of Russian LCs in the Watkins population, it can be speculated that this may have been brought about by Vavilov’s breeding efforts in the 1920s (Vavilov 1931). Watkins was inspired by Vavilov and presumably thus particularly acquired accessions from that region. It would be interesting to analyse the population structure of the IPK landrace collection (Huang et al. 2002), as it contains more Russian accessions than the Watkins collection, and possibly further groupings might be discovered.

The identification of the ancestral populations of the Watkins collection was made difficult by a noisy population structure. In the initial non-hierarchical approach, the nine ancestral populations were not detected. The presence of a weak signal of population structure suggests that the difference between groups was not strong. An exchange of breeding material between farmers on a larger scale may be one reason for the absence of a strong population structure. This may have overridden some of the signals of ancestral groupings. It would be interesting to line groupings up with predominant old trade routes, if that information was available. In addition, the accessions in the Watkins collection are not random samples, but were selected possibly based on geographic distribution and availability. This selection will have had an influence on the observed population structure, which was calculated assuming a random selection of LCs. Strong signals of population structure have been reported between wheat LCs, if the selection of cultivars came from geographically distinct places, e.g. in a comparison of LCs from Mexico and Turkey (Dreisigacker et al. 2005) and LCs from Turkey and Kazakhstan (Sayar-Turet et al. 2011). Due to the pre-selection of these LCs, the observed population structure is artificially high. It seems to be much stronger than the one we find in the broader, geographically more evenly spaced selection present in the Watkins collection. However, the weak population structure signal seems more realistic, as it is based on many more LCs. Overall, the detection of ancestral groupings means that different sets of gene combinations are present. This is more reason to expect new and useful alleles to be present in the collection. These alleles would have been conserved under some growing conditions, but would have been lost in the streams leading to the development of modern germplasm.

The Watkins collection is proven to host interesting alleles, particularly for disease resistance (Dyck 1994; Bansal et al. 2011; Thompson and Seymour 2011), which are mainly Mendelian genes and comparatively easy to identify. In order to facilitate the use of the collection, particularly for more complicated phenotypic screens, a core set of 119 lines was defined. This core set captures the majority of the genetic diversity and has been put to use by physiologists and breeders within the Wheat Improvement Strategic Programme (WISP, http://www.wheatisp.org).

Furthermore, a larger number of bi-parental mapping populations is under development, which will allow QTL mapping to be carried out. Transgressive segregants were discovered in several of these populations when scored for common traits. Furthermore, a number of QTLs have been identified. These observations and findings support the claim that the bi-parental populations will be valuable resources in the identification of new alleles in the wheat genome.

World wheat yields have plateaued in the last 10 years. All possible efforts are necessary to achieve a sufficient increase in world wheat yields, to keep up with the world’s growing human population and the challenges climate change will pose for agriculture. The detailed genotypic description of the Watkins LCs collection and the development of the new breeding resources should ultimately improve and accelerate allele discovery for simple, as well as for complex, traits and hopefully help to achieve the successful improvement of bread wheat, in line with the growing demand.

## Electronic supplementary material

Below is the link to the electronic supplementary material.

Supplementary material 1 (pdf 266 KB)
