# Supplementary material for: Establishing the A. E. Watkins landrace cultivar collection as a resource for systematic gene discovery in bread wheat
Source: Theor Appl Genet. 2014 Jul 2;127(8):1831–42. doi: 10.1007/s00122-014-2344-5 (PMC4110413; doi:10.1007/s00122-014-2344-5)
Supplement: Supplementary file 1 — Supplementary material 1 (pdf 266 KB) [file 122_2014_2344_MOESM1_ESM.pdf]

Theoretical Applied Genetics manuscript No.
(will be inserted by the editor)

Establishing the A. E. Watkins landrace cultivar collection as a resource for systematic gene discovery in bread wheat.

Luzie U. Wingen · Simon Orford · Richard Goram · Michelle Leverington-Waite, · Lorelei Bilham · Theofania S. Patsiou · Mike Ambrose · Jo Dicks · Simon Griffiths

Received: date / Accepted: date

References

[Anderson et al., 1993] Anderson, J. A., Churchill, G. A., Autrique, J. E., Tanksley, S. D., and Sorrells, M. E. (1993). Optimizing parental selection for genetic linkage maps. *Genome*, 36:181–186.

[Balfourier et al., 2007] Balfourier, F., Roussel, V., Strelchenko, P., Exbrayat-Vinson, F., Sourdille, P., Boutet, G., Koenig, J., Ravel, C., Mitrofanova, O., Beckert, M., and Charmet, G. (2007). A worldwide bread wheat core collection arrayed in a 384-well plate. *Theoretical and Applied Genetics*, 114:1265–1275.

[Huang et al., 2002] Huang, X. Q., Börner, A., Röder, M. S., and Ganal, M. W. (2002). Assessing genetic diversity of wheat (*Triticum aestivum* L.) germplasm using microsatellite markers. *Theoretical and Applied Genetics*, 105(5):699–707.

[Petit et al., 1998] Petit, R., El Mousadik, A., and Pons, O. (1998). Identifying populations for conservation on the basis of genetic markers. *Conserv Biol*, 12:844–855.

[Raman et al., 2010] Raman, H., Stodart, B., Cavanagh, C., Mackay, M., Morell, M., Milgate, A., and Martin, P. (2010). Molecular diversity and genetic structure of modern and traditional landrace cultivars of wheat (*Triticum aestivum* L.). *Crop. Pasture Sci.*, 61:222–229.

[Reeves et al., 2004] Reeves, J., Chiapparino, E., Donini, P., Ganal, M., Guiard, J., Hamrit, S., Heckenberger, M., Huan, X., Van, Kaauwen, M., Kochieva, E., Koebner, R., Law, J., Lea, V., LeClerc, V., Van der Lee, T., Leigh, F., Van der Linden, G., Malysheva, L., Melchinger, A., Orford, S., Reif, J., Röder, M., Schulman, A., Vosman, B., Van der Wiel, C., Wolf, M., and Zhang, D. (2004). Changes over time in the genetic diversity of four major European crops: a report from the GEDIFLUX Framework 5 Project. In Vollmann, J., Grausgruber, H., and Ruckenbauer, P., editors, *Genetic variation for plant breeding. Proceedings of the 17th EUCARPIA General Congress, Tulln, Austria, 8-11 September 2004*, pages 3–7.

[Reif et al., 2005] Reif, J. C., Melchinger, A. E., and Frisch, M. (2005). Genetical and mathematical properties of similarity and dissimilarity coefficients applied in plant breeding and seed bank management. *Crop Science*, 45:1–7.

[Röder et al., 2002] Röder, M., Wendehake, K., Korzun, V., Bredemeijer, G., Laborie, D., Bertrand, L., Isaac, P., Rendell, S., Jackson, J., Cooke, R., Vosman, B., and Ganal, M. (2002). Construction and analysis of a microsatellite-based database of European wheat varieties. *Theor Appl Genet.*, 106(1):67–73.

[Roussel et al., 2005] Roussel, V., Leisova, L., Exbayat, F., and Balfourier, F. (2005). SSR allelic diversity changes in 480 European bread wheat varieties released from 1840 to 2000. *Theoretical and Applied Genetics*, 111:162–170.

L.U. Wingen, S. Orford, R. Goram, M. Leverington-Waite, L. Bilham, M. Ambrose, T. S. Patsiou, J. Dicks, S. Griffiths
John Innes Centre
Norwich Research Park
Norwich, UK
Tel.: +1603-450-508
Fax: +1603-450-045
E-mail: luzie.wingen@jic.ac.uk

[Thachuk et al., 2009] Thachuk, C., Crossa, J., Franco, J., Dreisigacker, S., Warburton, M., and Davenport, G. F. (2009). Core hunter: an algorithm for sampling genetic resources based on multiple genetic measures. *BMC Bioinformatics*, 10(243).

[Wei et al., 2005] Wei, Y.-M., Hou, Y.-C., Yan, Z.-H., Wu, W., Zhang, Z.-Q., Liu, D.-C., and Zheng, Y.-L. (2005). Microsatellite DNA polymorphism divergence in Chinese wheat (*Triticum aestivum* L.) landraces highly resistant to fusarium head blight. *Journal of Applied Genetics*, 46:3–9.

[White et al., 2008] White, J., Law, J., MacKay, I., Chalmers, K., Smith, J., Kilian, A., and Powell, W. (2008). The genetic diversity of UK, US and Australian cultivars of *Triticum aestivum* measured by DArT markers and considered by genome. *Theoretical and Applied Genetics*, 116:439–453.

List of Figures

1 **Supplementary Material: A**: lnPD statistic of the main STRUCTURE run, **B-D**:δK statistics to determine the number of ancestral populations in the Watkins collection: complete collection (B), 424 subgroup (C), and 630 subgroup (D). 4

2 **Supplementary Material: A**: lnPD statistic of the main STRUCTURE run on Gediflux collection, **B**:δK statistics to determine the number of ancestral populations whole collection (B) 5

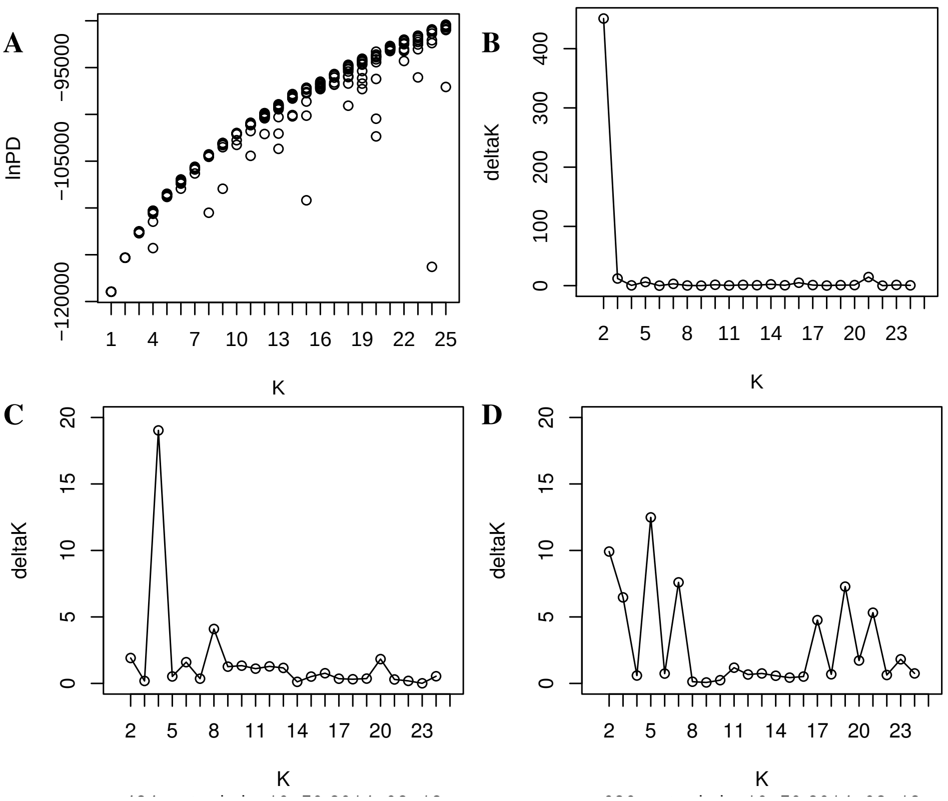

Fig. 1 Supplementary Material: A: lnPD statistic of the main STRUCTURE run, **B-D**:δK statistics to determine the number of ancestral populations in the Watkins collection: complete collection (B), 424 subgroup (C), and 630 subgroup (D).

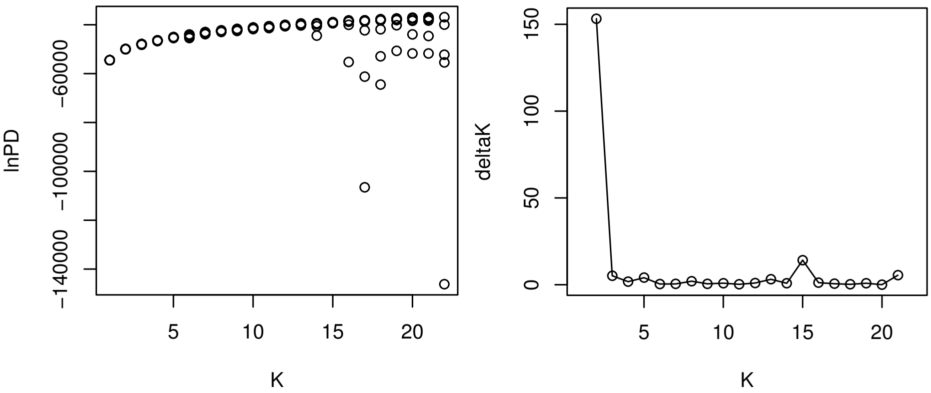

Fig. 2 Supplementary Material: A: lnPD statistic of the main STRUCTURE run on Gediflux collection, **B**:δK statistics to determine the number of ancestral populations whole collection (B)

List of Tables

1 **Supplementary Material:** Common genetic diversity indices. n is the total number of allele occurrences. n_i is the number of occurrences of the ith allele. g is the number of loci. $n > g$. n_{st} is the total number of allele occurrences in a standardised set, all sets of similar size. g_{st} is the number of loci in a standardised set. $n_{st} > g_{st}$. p_i is the frequency of the ith allele. p_{ij} and q_{ij} are allele frequencies of the jth allele at the ith locus. For original citations see [Petit et al., 1998] for eq. 3; [Anderson et al., 1993] for eq 4; [Reif et al., 2005] for eqs. 5,6, and 7; and [Thachuk et al., 2009] for eq. 8. . 8

2 **Supplementary Material:** Summary statistics of the phenotype values observed for 726 LCs of the Watkins collection in field trials in 2006. Names of LCs that were selected as a parent for the development of bi-parental populations are listed in the last two columns. The selection criterion for the LC parent was the display of a phenotype within the extreme borders of the phenotype range. Broad-sense heritability for plant height was 0.92 and for days to heading date 0.98. Abbreviation: d_{SWIp}=Shannon-Weaver Diversity Index, calculated on phenotypic classes. 9

3 **Supplementary Material:** Summary statistics of the phenotypic values observed for the Gediflux collection (top) and for the Watkins core set (119 LCs) in 2011 (bottom). Abbreviations: min=minimal phenotypic value, max=maximal phenotypic value, H^2=broad-sense heritability, d_{SWIp}=Shannon-Weaver diversity index, calculated on phenotypic classes, V_g=genotypic variance, V_e=environmental variance, W2006= Watkins collection grown in 2006 (for comparison) 10

4 **Supplementary Material:** Summary of the genotyping outcome [Reeves et al., 2004] and diversity statistics for 42 SSR markers on the Gediflux collection. Abbreviations: chr: putative chromosome locations according to Gramene database (http://www.gramene.org/markers), d_{AR}: Allele Richness, d_{RAR}: Number Of Rare Alleles, $\hat{r}(g)$: Allele Richness After Rare-faction, d_{Nei}: Nei's Gene Diversity, d_{PIC}: Polymorphic Information Content, d_{SWI}: Shannon-Weaver Diversity Index See Tab. 1 for the diversity index keys in the column headers. 11

5 **Supplementary Material:** List of accessions and country of origin of LCs included in the core set (119 LCs). 12

6 **Supplementary Material:** Diversity statistics of the core set (119 LCs). The core set preserves 98% or 96% of the total genetic diversity (diversity index d_{Nei} or d_{SWI}, respectively) 13

7 **Supplementary Material:** Average levels of genetic diversity of wheat collections as published in the literature compared to the present study. Abbreviations: LC landrace cultivar; MC modern elite cultivar; UK United Kingdom; USA United States of America; AUS Australia; EUR Europe; CHI China; IPK Leibniz Institute of Plant Genetics and Crop Plant Research, Germany; INRA French National Institute for Agricultural Research; n.a. not applicable 14

8 **Supplementary Material:** Genetic markers employed on the Watkins collection that were also used by [Huang et al., 2002] (H) and/or [Balfourier et al., 2007] (B) and the detected levels of genetic diversity for these markers. 15

9 **Supplementary Material:** Comparison of the diversity statistics for 14 markers used to genotype the Watkins collection as well as the Gediflux collection. Abbreviations: chr: chromosome locations according to Gramene database (http://www.gramene.org/markers). See Tab. 1 for the diversity index keys in the column headers. Columns labelled G for Gediflux collection or W for Watkins collection. 16

Table 1 Supplementary Material: Common genetic diversity indices. n is the total number of allele occurrences. n_i is the number of occurrences of the ith allele. g is the number of loci. $n > g$. n_{st} is the total number of allele occurrences in a standardised set, all sets of similar size. g_{st} is the number of loci in a standardised set. $n_{st} > g_{st}$. p_i is the frequency of the ith allele. p_{ij} and q_{ij} are allele frequencies of the jth allele at the ith locus. For original citations see [Petit et al., 1998] for eq. 3; [Anderson et al., 1993] for eq 4; [Reif et al., 2005] for eqs. 5,6, and 7; and [Thachuk et al., 2009] for eq. 8.

genetic diversity index	equation	
Allele Richness (d_{AR})	n	
Average Allele Number	$\frac{n}{g}$	(1)
Standardised Number Of Alleles	$\frac{n_{st}}{g_{st}}$	(2)
Number Of Rare Alleles (d_{RAR})	number of alleles with frequency $< 2\%$	
Allele Richness After Rare-faction ($\hat{r}(g)$)	$\hat{r} = \sum_{i=1}^{g} \binom{n-n_i}{g} \binom{n}{g}$	(3)
Polymorphic Information Content (d_{PIC})	$d_{PIC} = 1 - \sum_{i}^{g}(p_i)^2 - \sum_{i}^{g} \sum_{j=i+1}^{g} 2p_i^2 p_j^2$	(4)
Nei's Gene Diversity (d_{Nei})	$d_{Nei} = 1 - \sum_{i}^{g}(p_i)^2$	(5)
Modified Rogers Distance (d_{MR})	$d_{MR} = \frac{1}{\sqrt{2g}} \sqrt{\sum_{i=1}^{g} \sum_{j=1}^{n_j} (p_{ij} - q_{ij})^2}$	(6)
Cavalli-Sforza and Edwards Distance (d_{CE})	$d_{CE} = \sqrt{\frac{1}{g} \sum_{i=1}^{g} (1 - \sum_{j=1}^{n_j} \sqrt{p_{ij} q_{ij}})}$	(7)
Shannon-Weaver Diversity Index (d_{SWI})	$d_{SWI} = -\sum_{i}^{g}(p_i ln p_i)$	(8)

Table 2 Supplementary Material: Summary statistics of the phenotype values observed for 726 LCs of the Watkins collection in field trials in 2006. Names of LCs that were selected as a parent for the development of bi-parental populations are listed in the last two columns. The selection criterion for the LC parent was the display of a phenotype within the extreme borders of the phenotype range. Broad-sense heritability for plant height was 0.92 and for days to heading date 0.98. Abbreviation: d_{SWIp}=Shannon-Weaver Diversity Index, calculated on phenotypic classes.

trait	units	mean	sd	min	max	d_{SWIp}	LCs near max	LCs near min
plant height	cm	99.6	13.0	49.5	139.8	1.71	1190141<br>1190352	1190292<br>1190729
heading date	days	18.1	9.0	0.0	51.2	1.96	1190034<br>1190209<br>1190468	
thousand grain weight	g	34.9	6.2	17.1	53.5	1.64	1190238	1190308<br>1190732<br>1190777
grain surface area	cm^2	18.9	1.8	13.4	26.7	2.03	1190103<br>1190627<br>1190680	1190827
grain width	cm	3.4	0.2	2.8	3.9	1.37	1190548	1190299<br>1190300<br>1190475
grain length	cm	6.6	0.5	5.1	8.8	1.39	1190627<br>1190629	1190811

Table 3 Supplementary Material: Summary statistics of the phenotypic values observed for the Gediflux collection (top) and for the Watkins core set (119 LCs) in 2011 (bottom). Abbreviations: min=minimal phenotypic value, max=maximal phenotypic value, H^2=broad-sense heritability, d_{SWIp}=Shannon-Weaver diversity index, calculated on phenotypic classes, V_g=genotypic variance, V_e=environmental variance, W2006= Watkins collection grown in 2006 (for comparison)

.

	trait	units	mean	sd	min	max	H^2	d_{SWIp}	$V_g \pm$ SD	$V_e \pm$ SD
Gediflux	plant height	cm	66.3	11.6	45.0	112.8	0.97	1.47	125 ±11	12.7 ±3.6
	heading date	days	22.8	3.9	9.7	33.3	0.96	1.20	134 ±1.2	1.8 ±1.4
	grain yield	t/ha	10.5	1.6	3.9	14.5	0.82	-	200 ±14	133±12
	thousand grain weight	g	51.9	4.5	38.6	69.4	0.88	1.35	17.1 ±4.1	7.1 ±2.7
	grain surface area	cm^2	23.1	1.4	19.1	27.7	0.92	1.74	1.7 ±1.3	0.4 ±0.7
	grain width	cm	4.2	0.1	3.7	4.5	0.87	1.08	0.0 ±0.1	0.0 ±0.1
	grain length	cm	6.9	0.3	6.2	7.9	0.95	0.97	0.1 ±0.3	0.0 ±0.1
	ear length	cm	8.0	0.8	5.8	10.9	0.90	-	2.3 ±1.5	0.7 ± 0.9
	spikelets per ear		21.0	1.9	14.5	31.9	0.82	-	1.6 ±1.3	1.1 ±1.0
	fertile spikelets per ear		17.7	1.6	12.4	25.3	0.97	-	24.8 ±5.0	2.1 ±1.4
	peduncle length	cm	20.4	5.2	11.6	39.1	0.92	-	2.9 ±1.7	0.8 ±0.9
	internode 1 length	cm	11.0	2.0	7.1	18.6	0.90	-	1.2 ±1.1	0.4 ±0.6
	internode 2 length	cm	7.0	1.3	4.0	13.1	0.90	-	0.7 ±0.8	0.2 ±0.5
	internode 3 length	cm	5.3	1.0	2.9	9.7	0.91	-	0.6 ±0.8	0.2 ±0.5
	internode 4 length	cm	4.0	0.9	2.2	8.5	0.83	-	0.3 ±0.5	0.2 ±0.4
	internode 5 length	cm	1.7	0.6	0.5	4.3	0.44	-	0.0 ±0.1	0.1 ±0.2
	internode 6 length	cm	0.7	0.3	0.3	2.5	0.75	-	0.1 ±0.2	0.1 ±0.2
	internode mean length	cm	6.0	0.9	4.0	9.7	0.90	-	0.4 ±0.6	0.1 ±0.4
	internode number	cm	4.7	0.3	3.0	5.6	0.91	-	0.7 ±0.8	0.2 ±0.4
	sd internode mean length	cm	3.5	0.7	2.0	6.2	0.49	-	0.0 ±0.1	0.0 ±0.2
Watkins Core	heading date	days	16.0	5.7	5.0	31.0	-	1.58	-	-
	grain yield	t/ha	2.7	0.8	0.3	5.6	-	-	-	-
	thousand grain weight	g	46.6	8.7	23.8	73.4	-	1.92	-	-
	grain surface area	cm^2	19.9	2.3	14.1	27.8	-	2.15	-	-
	grain width	cm	3.7	0.2	3.1	4.3	-	1.49	-	-
	grain length	cm	6.8	0.5	5.4	8.8	-	1.48	-	-
W2006	heading date	days	18.1	9.0	0.0	51.2	0.98	1.96	76.3 ±8.7	7.4 ±2.7
	plant height	cm	99.6	13.0	49.5	139.8	0.92	1.71	151 ±12	52.7 ±7.3

Table 4 Supplementary Material: Summary of the genotyping outcome [Reeves et al., 2004] and diversity statistics for 42 SSR markers on the Gediflux collection. Abbreviations: chr: putative chromosome locations according to Gramene database (http://www.gramene.org/markers), d_{AR}: Allele Richness, d_{RAR}: Number Of Rare Alleles, $\hat{r}(g)$: Allele Richness After Rare-faction, d_{Nei}: Nei's Gene Diversity, d_{PIC}: Polymorphic Information Content, d_{SWI}: Shannon-Weaver Diversity Index See Tab. 1 for the diversity index keys in the column headers.

marker name	chr	missing	d_{AR}	d_{RAR}	$\hat{r}(g)$	d_{Nei}	d_{PIC}	d_{SWI}
gwm003	3D	4.7	4	2	2.3	0.50	0.38	0.72
gwm018	1B	0.2	10	3	5.3	0.62	0.54	1.21
gwm046	7B	0.2	12	3	7.8	0.69	0.67	1.61
gwm095	2A	1.2	6	1	4.8	0.63	0.58	1.22
gwm155	3A	1.6	9	3	5.0	0.57	0.51	1.13
gwm190	5D	0.4	7	2	5.0	0.74	0.70	1.46
gwm213	5B	0.4	14	8	6.4	0.70	0.66	1.50
gwm219	6B	2.0	16	8	8.3	0.78	0.76	1.85
gwm261	2D	0.0	7	3	4.0	0.46	0.42	0.89
gwm272	5D	33.3	5	0	4.4	0.66	0.60	1.22
gwm291	5A	3.3	11	4	6.3	0.65	0.62	1.42
gwm312	2A	0.4	19	9	9.2	0.79	0.77	1.95
gwm320	2D	1.8	9	2	6.7	0.73	0.70	1.58
gwm325	6D	55.8	6	3	3.7	0.57	0.48	0.99
gwm334	6A	0.6	8	1	5.8	0.66	0.60	1.34
gwm357	1A	0.6	4	1	3.1	0.53	0.47	0.92
gwm389	3B	0.8	12	4	7.6	0.80	0.77	1.82
gwm408	5B	1.2	11	6	5.0	0.64	0.57	1.24
gwm415	5A	57.5	2	0	1.5	0.03	0.03	0.07
gwm437	7D	0.2	17	11	6.4	0.53	0.50	1.23
gwm456	3D	4.3	9	3	5.8	0.70	0.65	1.44
gwm458	1D	33.1	3	0	2.7	0.48	0.39	0.75
gwm513	4B	0.0	5	1	3.7	0.32	0.30	0.67
gwm570	6A	7.8	9	2	6.0	0.68	0.63	1.40
gwm577	7B	1.4	23	11	11.9	0.88	0.87	2.39
gwm610	4A	0.0	7	4	3.4	0.19	0.18	0.46
gwm619	2B	0.0	11	5	6.4	0.73	0.69	1.56
gwm631	7A	0.6	7	3	3.7	0.54	0.44	0.92
gwm680	6B	0.0	7	5	2.6	0.52	0.40	0.79
gwm793	1B	8.4	8	1	6.5	0.77	0.73	1.64
gwm818	1B	1.2	10	5	5.7	0.70	0.66	1.45
gwm819	4D	16.6	3	0	2.7	0.19	0.18	0.39
gwm834	7A	0.2	16	6	8.9	0.70	0.68	1.73
gwm1397		9.4	17	7	9.8	0.83	0.82	2.09
gwm1531		0.4	11	3	5.7	0.47	0.44	1.05
gwm1691		0.6	8	3	4.9	0.56	0.52	1.14
gwm1749		0.4	26	13	12.1	0.83	0.82	2.28
gwm1750		0.8	12	2	7.1	0.67	0.61	1.46
gwm3072	4B	17.6	8	2	5.4	0.68	0.63	1.36
gwm3092		2.0	14	5	8.3	0.70	0.68	1.69
gwm3094		0.2	16	9	7.5	0.79	0.76	1.79
gwm3144	3B	8.8	15	7	7.4	0.67	0.64	1.53
mean		6.7	10.3	4.1	5.9	0.62	0.57	1.30
min		0.0	2	0	1.5	0.03	0.03	0.07
max		57.5	26	13	12.1	0.88	0.87	2.39

Table 5 Supplementary Material: List of accessions and country of origin of LCs included in the core set (119 LCs).

accession name	country code	country of origin	remarks
1190004	IRQ	Iraq	Rustam Exp Farm 99
1190007	AUS	Australia	
1190023	AUS	Australia	College Purple
1190032	IND	India	Dehak
1190034	IND	India	Desi
1190040	FRA	France	Favori
1190042	FRA	France	Inversal
1190044	MAR	Morocco	
1190045	SYR	Syria	Douchani
1190079	IND	India	Dolatkhani (white)
1190081	IND	India	Gahu (Nepali) or Kyo (Sikkimese)
1190092	IND	India	Desi
1190103	ITA	Italy	Gentil Rossa
1190110	FRA	France	Carré Géant blanc
1190126	IND	India	Dolatkhani (white)
1190127	IND	India	Thori (beardless)
1190139	FRA	France	Bladette de Besplas
1190141	CHN	China	
1190145	ESP	Spain	Aurora
1190149	GBR	United Kingdom	Squarehead's Master
1190160	ESP	Spain	Mocho de Burgos
1190166	IND	India	Meerutti Desi
1190181	POL	Poland	Kujawianka Wieclawicka
1190199	IND	India	Boojri (bearded)
1190209	EGY	Egypt	Belardi 24B
1190216	MAR	Morocco	
1190218	TUN	Tunisia	Sbei Noir
1190219	ESP	Spain	
1190223	MMR	Burma	Shan wheat
1190224	CHN	China	Red wheat
1190231	HUN	Hungary	
1190238	IRN	Iran	
1190239	ESP	Spain	Barbilla
1190246	IND	India	Soor Ghanum
1190254	MAR	Morocco	
1190264	CAI	Canary Islands	
1190273	ESP	Spain	
1190281	GRC	Greece	Deve
1190291	CYP	Cyprus	Psathas
1190292	CYP	Cyprus	Asprokoutsoullon
1190299	TUR	Turkey	
1190300	TUR	Turkey	
1190305	EGY	Egypt	
1190308	IRN	Iran	
1190313	MMR	Burma	2193/20-12
1190324	CHN	China	
1190325	GBR	United Kingdom	
1190349	BGR	Bulgaria	Golema Franga
1190352	YUG	Yugoslavia	
1190355	YUG	Yugoslavia	
1190360	YUG	Yugoslavia	Veliko Hoce
1190387	ESP	Spain	
1190396	PRT	Portugal	Trigo Rietti
1190397	PRT	Portugal	Trigo Ribeiro
1190398	PLE	Palestine	Abu Fashi
1190406	IND	India	Desi
1190420	IND	India	Dhania
1190433	IND	India	Soor Ghanum
1190440	CHN	China	
1190444	CHN	China	
1190451	ROU	Romania	Samanta 117
1190460	AFG	Afghanistan	
1190468	AFG	Afghanistan	
1190471	AFG	Afghanistan	
1190474	AFG	Afghanistan	
1190475	AFG	Afghanistan	
1190481	POL	Poland	Ostka Skomoroska
1190483	POL	Poland	Surka Oscista
1190496	MAR	Morocco	
1190507	AUS	Australia	
1190546	ESP	Spain	Hembrilla alto
1190551	ESP	Spain	Hembrilla Rieti
1190560	GRC	Greece	Asprotheri
1190562	GRC	Greece	
1190566	GRC	Greece	
1190568	CHN	China	
1190579	IRN	Iran	Vassfe
1190580	IRN	Iran	
1190591	PRT	Portugal	Trigo Anafil
1190605	GRC	Greece	Karabash
1190624	BGR	Bulgaria	Svalene
1190627	IRN	Iran	
1190629	IRN	Iran	Qizilqin Boghda
1190637	TUR	Turkey	
1190639	CRE	Crete	
1190651	CHN	China	
1190652	CHN	China	
1190662	ROU	Romania	Samanta 1252
1190670	POL	Poland	Zlotka Miczynskiego
1190671	SUN	USSR	ferrugineum Al.
1190680	ITA	Italy	Grano locale
1190683	ESP	Spain	Catalan de grano corto pesado
1190685	ESP	Spain	Trigo duros
1190690	GRC	Greece	
1190694	IND	India	Lyallpur 8A
1190698	CHN	China	
1190700	CHN	China	Kaifeng 323-9
1190704	IRN	Iran	Ma'auneh
1190705	IRN	Iran	Kooseh
1190707	IND	India	Walaiti (Mastung wheat)
1190722	CHN	China	
1190729	IRN	Iran	Gandum-i-Jiruft
1190731	IND	India	Gahu (Nepali) or Kyo (Sikkimese)
1190732	IND	India	Gahu (Nepali) or Kyo (Sikkimese)
1190740	SUN	USSR	
1190742	DZA	Algeria	
1190746	SUN	USSR	Muganka
1190747	ETH	Ethiopia	
1190749	SUN	USSR	
1190750	SUN	USSR	Valki Exp Sta 3
1190753	SUN	USSR	Valki Exp Sta 1
1190771	SUN	USSR	
1190777	FIN	Finland	
1190784	ITA	Italy	Oberdan
1190788	SUN	USSR	
1190789	SUN	USSR	Abi
1190811	TUN	Tunisia	
1190814	TUN	Tunisia	Allorca
1190816	ITA	Italy	
1190827	CHN	China	
1190912	HUN	Hungary	

Table 6 Supplementary Material: Diversity statistics of the core set (119 LCs). The core set preserves 98% or 96% of the total genetic diversity (diversity index d_{Nei} or d_{SWI}, respectively)

marker name	missing	d_{AR}	d_{RAR}	$\hat{r}(g)$	d_{Nei}	d_{PIC}	d_{SWI}
barc019	0.80	12	4	8.20	0.77	0.74	1.78
barc021	2.50	11	3	7.80	0.57	0.55	1.38
barc029	3.30	7	1	5.70	0.68	0.63	1.36
barc032	0.80	11	1	9.50	0.84	0.82	2.04
barc096	0.80	5	2	3.60	0.51	0.44	0.89
barc097	18.20	7	0	5.70	0.75	0.71	1.52
barc107	1.70	9	4	5.40	0.54	0.48	1.08
barc110	0.80	20	5	15.10	0.92	0.92	2.71
barc127	0.80	9	2	7.50	0.81	0.78	1.82
barc134	16.50	14	5	9.30	0.80	0.78	1.93
barc164	0.80	19	5	13.30	0.88	0.87	2.45
barc172	0.80	9	0	7.50	0.80	0.77	1.78
barc240	0.80	23	10	13.90	0.86	0.85	2.44
cfd079.a	9.10	19	4	14.00	0.90	0.90	2.56
cfd079.b	14.90	5	0	4.90	0.69	0.64	1.35
gdm111.b	7.40	10	2	7.60	0.77	0.74	1.73
gdm129	0.80	6	3	3.80	0.20	0.20	0.49
gwm003	0.80	11	2	8.00	0.72	0.69	1.66
gwm018	1.70	8	0	7.00	0.75	0.71	1.62
gwm030.a	28.90	11	2	8.50	0.77	0.75	1.80
gwm030.b	9.90	21	3	17.30	0.94	0.93	2.88
gwm046	4.10	19	3	14.20	0.91	0.90	2.58
gwm095	0.80	8	1	7.00	0.82	0.80	1.83
gwm155	0.80	15	3	10.40	0.79	0.77	1.97
gwm190	0.80	13	5	8.90	0.81	0.79	1.93
gwm213	1.70	36	11	23.00	0.95	0.95	3.31
gwm219	0.80	23	5	17.00	0.93	0.92	2.83
gwm291	12.40	22	4	16.00	0.88	0.87	2.59
gwm312	0.80	27	10	15.90	0.88	0.87	2.61
gwm337	3.30	19	4	13.10	0.86	0.85	2.38
gwm357	1.70	6	1	4.90	0.70	0.64	1.34
gwm437	0.80	22	4	16.40	0.92	0.92	2.78
gwm456	1.70	15	5	10.50	0.83	0.81	2.08
gwm526.a	4.10	4	0	4.00	0.62	0.57	1.16
gwm526.b	3.30	10	2	7.70	0.74	0.71	1.66
gwm539	1.70	36	16	21.80	0.95	0.95	3.24
gwm570	0.80	19	6	13.40	0.90	0.89	2.50
gwm608.a	20.70	6	0	6.00	0.79	0.76	1.68
gwm608.c	0.80	13	1	11.00	0.89	0.88	2.33
psp3100	1.70	34	14	19.60	0.93	0.92	3.02
wmc093	1.70	5	2	2.90	0.51	0.40	0.80
wmc105	3.30	30	12	17.20	0.91	0.90	2.80
wmc110	28.90	3	0	2.70	0.27	0.24	0.50
wmc154	0.80	22	9	13.00	0.76	0.75	2.12
wmc168	14.00	27	9	16.40	0.84	0.84	2.54
average	5.20	15.10	4.10	10.60	0.77	0.75	2.00
min	0.8	3	0	2.7	0.20	0.20	0.49
max	28.9	36	16	23	0.95	0.95	3.31

Table 7 Supplementary Material: Average levels of genetic diversity of wheat collections as published in the literature compared to the present study. Abbreviations: LC landrace cultivar; MC modern elite cultivar; UK United Kingdom; USA United States of America; AUS Australia; EUR Europe; CHI China; IPK Leibniz Institute of Plant Genetics and Crop Plant Research, Germany; INRA French National Institute for Agricultural Research; n.a. not applicable

material	accession number	marker number	average allele number	range of allele number	d_{PIC}	Reference
IPK LCs	998	24	18.1	4-46	0.77	[Huang et al., 2002]
MCs	502	20	10.5	4-22	0.65	[Röder et al., 2002]
CHI LCs	24	40	6.9	1-16	0.42	[Wei et al., 2005]
EUR MCs 1840-2000	480	39	16.4	4-40	0.65	[Roussel et al., 2005]
UK/USA/AUS MCs 1845-2005	140	379	DArT	n. a.	0.40	[White et al., 2008]
INRA LCs + MCs	3,942	38	23.9	7-45	0.74	[Balfourier et al., 2007]
AUS LCs + MCs	1,057	178	DArT	n.a.	0.44	[Raman et al., 2010]
Gediflux EUR MCs 1945-2000	511	42	10.5	1-16	0.65	this study
Watkins LCs	826	41	22.4	3-65	0.75	this study

Table 8 **Supplementary Material:** Genetic markers employed on the Watkins collection that were also used by [Huang et al., 2002] (H) and/or [Balfourier et al., 2007] (B) and the detected levels of genetic diversity for these markers.

marker	chr	d_{AR}	d_{RAR}	d_{Nei}	d_{AR} (H)	d_{Nei} (H)	d_{AR} (B)	d_{RAR} (B)	d_{Nei} (B)
gwm003	3D	16	9	0.69	10	0.67	-	-	-
gwm018	1B	15	7	0.74	13	0.77	-	-	-
gwm046	7B	28	10	0.91	24	0.88	27	21	0.87
gwm095	2A	14	7	0.81	13	0.78	-	-	-
gwm190	5D	22	12	0.84	23	0.82	25	19	0.74
gwm219	6B	16	8	0.78	-	-	30	24	0.87
gwm312	2A	19	9	0.79	-	-	45	39	0.87
gwm325	6D	6	3	0.57	-	-	18	11	0.77
gwm337	1D	25	11	0.88	24	0.83	23	19	0.85
gwm357	1A	11	4	0.73	12	0.69	-	-	-
gwm437	7D	26	8	0.93	23	0.90	28	22	0.86
gwm539	2D	61	34	0.96	-	-	40	35	0.89

Table 9 Supplementary Material: Comparison of the diversity statistics for 14 markers used to genotype the Watkins collection as well as the Gediflux collection. Abbreviations: chr: chromosome locations according to Gramene database (http://www.gramene.org/markers). See Tab. 1 for the diversity index keys in the column headers. Columns labelled G for Gediflux collection or W for Watkins collection.

marker name	chr	d_{AR} G	d_{RAR} G	$\hat{r}(g)$ G	d_{Nei} G	d_{PIC} G	d_{SWI} G	d_{AR} W	d_{RAR} W	$\hat{r}(g)$ W	d_{Nei} W	d_{PIC} W	d_{SWI} W
gwm003	3D	4	2	2.3	0.50	0.38	0.72	16	9	7.2	0.69	0.66	1.56
gwm018	1B	10	3	5.3	0.62	0.54	1.21	15	7	7.8	0.74	0.71	1.69
gwm046	7B	12	3	7.8	0.69	0.67	1.6	28	10	15.1	0.91	0.91	2.71
gwm095	2A	6	1	4.8	0.63	0.58	1.22	14	7	7.7	0.81	0.79	1.86
gwm155	3A	9	3	5.0	0.57	0.51	1.13	24	13	10.1	0.80	0.78	2.01
gwm190	5D	7	2	5.0	0.74	0.70	1.46	22	12	9.9	0.84	0.83	2.12
gwm213	5B	14	8	6.4	0.70	0.66	1.50	46	19	23	0.96	0.95	3.38
gwm219	6B	16	8	8.3	0.78	0.76	1.85	30	11	16.0	0.91	0.91	2.76
gwm291	5A	11	4	6.3	0.65	0.62	1.42	29	12	15.0	0.87	0.86	2.56
gwm312	2A	19	9	9.2	0.79	0.77	1.95	43	25	17.3	0.89	0.88	2.80
gwm357	1A	4	1	3.1	0.53	0.47	0.92	11	4	6.1	0.73	0.69	1.53
gwm437	7D	17	11	6.4	0.53	0.50	1.23	26	8	16.3	0.93	0.93	2.83
gwm456	3D	9	3	5.8	0.70	0.65	1.44	18	7	10.1	0.84	0.82	2.10
gwm570	6A	9	2	6.0	0.68	0.63	1.40	26	14	12.7	0.89	0.88	2.45
